# Comparative Analysis of the Chemical Composition and Microstructure Conformation Between Different Dental Implant Bone Drills

**DOI:** 10.3390/ma12111866

**Published:** 2019-06-09

**Authors:** Gaetano Marenzi, Josè Camilla Sammartino, Fabio Scherillo, Carlo Rengo, Alfredo De Rosa, Vincenzo Graziano, Gianrico Spagnuolo

**Affiliations:** 1 Department of Neurosciences, Reproductive and Odontostomatological Sciences, University of Naples “Federico II”, Via Pansini 5, 80131 Naples, Italy; gaetano.marenzi@gmail.com; 2 Department of Biology and Biotechnology “Lazzaro Spallanzani”, University of Pavia Via Ferrata 9, 27100 Pavia, Italy; jose.sammartino@iusspavia.it; 3 Department of Chemical, Materials and Industrial Production Engineering, University of Naples “Federico II”, Piazzale Tecchio 80, 80125 Naples, Italy; fabio.scherillo@unina.it; 4 Department of Prosthodontics and Dental Materials, School of Dental Medicine, University of Siena, 53100 Siena, Italy; carlorengo@alice.it; 5 Department of Odontology and Surgery, University of Campania “L. Vanvitelli”, Via S. Andrea delle Dame 6, 80138 Naples, Italy; dinoderosa@live.it; 6 Department of Advanced Biomedical Sciences, University of Naples “Federico II”, Via Pansini 5, 80131 Naples, Italy; vincenzo.graziano2@unina.it; 7 Institute of Dentistry, I. M. Sechenov First Moscow State Medical University, Moscow 119146, Russia

**Keywords:** dental implant drill, microstructural surface analysis, bone drill chemical composition, drill hardness

## Abstract

Background: Hardness is considered an important parameter for evaluating the clinical performance of dental implant bone drills. It is connected to the chemical composition, microstructure conformation and manufacture of the surgical drills. Methods: Microstructure of five dental implant drills using scanning electronic microscopy (SEM) integrated with energy dispersive X-ray spectrometry. Vickers microhardness was measured using a CV 2000 microhardness tester with an indentation force of 500 g. Results: Composition of the implant drills was typical of martensitic stainless steel (MSS). The drills contained 13%–17% of Cr; Mo, Si and Mn were present as minor ligands. The examined bone drills showed different external surface conformation and hardness in relation to the different industrial production processes. A rougher external surface and a higher hardness value are characteristics of the surgical bone drills produced by hot forming; the implant drills produced by machining showed mailing lines on their external surface and a lower hardness. Conclusions: Different compositions and treatments were used by the manufacturers to improve the hardness of the external layer of the dental implant drills making them prone to a diverse heat generation during the implant site preparation.

## 1. Introduction

The success of dental implant therapy depends on many factors, several of which are influenced by the surgical technique used [1,2,3]. Atraumatic preparation of the osteotomy site is critical for predictability and enhanced osseointegration [4]. In recent decades, many authors have sought to identify factors that minimize the damage during implant site preparation, but there is no general agreement on the mechanical modeling of this process to determine optimum drill design and drill parameters for avoiding bone necrosis [4]. Indeed, even when appropriate care is taken during drilling procedures, a thin layer of necrotic bone tissue is always detected and the tissue in question is subsequently replaced by vital bone tissue [5]. All the surgical factors that increase thickness of the necrotic layer can negatively affect bone tissue maturation and compromise implant osseointegration [5]. Extension of the necrotic zone around the preparation site is considered proportional to the amount of heat generated by surgical drills during osteotomy, which can be related to different factors: (i) Operator (pressure, status, movement, speed and duration of drilling), (ii) manufacturer (design and sharpness of the drill, irrigation system), (iii) implant site (cortical thickness, bone density, depth drilled) and (iv) patient (age) [2,3,6,7,8,9,10,11,12,13,14,15,16,17,18,19]. Several authors have investigated the heat produced during osteotomy generated by different drill materials, accuracy of construction, efficiency of cutting and wear [20,21,22,23,24,25]. While the phenomenon of heat production during bone drilling is well known, the different potential for bone thermal damage during implant site preparation correlated to the diverse chemical composition and surface conformation is inconsistently reported in the literature. 

The aim of this study was to analyze the chemical composition and microstructural conformation of implant drills made by five commercial manufacturers, evaluating their predisposition to heat generation during dental implant placement.

## 2. Materials and Methods 

Implant drills approximately 2.0 mm in diameter were selected because they usually represent the first bone drills to be used for implant site preparation, drilling both cortical and cancellous bone. The following commercially available dental implant drills (five drills from each manufacturer) were analyzed in the present research:

- Straumann (Institut Straumann AG, Basel, Switzerland) 2.2 mm in diameter
- NobelBiocare (NobelBiocare AB, Gothenburg, Sweden) 2.0 mm in diameter
- Xive Implant System (Desplay Friadent GmbH, Mannheim, Germany,) 2.0 mm in diameter
- Global D (In-Kone GlobalD Universal, Brignais, France) 2.0 mm in diameter
- Sweden & Martina (Sweden & Martina, Due Carrare, Italy) 2.0 mm in diameter

Chemical and microstructural analysis was performed in the Department of Chemical, Materials and Industrial Production Engineering of the University of Naples “Federico II”. The comparative analysis was performed considering for each bone drill the cylindrical external surface at a distance of 1 mm to the cutting edges. At the same level the implant drills were cut by a metallographic power cutter for evaluating the chemical composition of the “core”. Then the implant drills were cold-embedded in epoxy resin and polished with a mirror finish with (a) P320 sandpaper, (b) 9 μm diamond suspension, (c) 3 μm diamond suspension and (d) 0.05 μm emulsion of colloidal silica. According to the international standard guide for the preparation of a metallographic specimen, the samples were then subjected to ultrasonic cleaning in distilled water and etching by means of Vilella reagent (a 10:1 mix solution of 4% Picral acid and 2% Nital acid in ethanol) for removing any kind of contaminant from the surfaces [26].

A surface morphology observation along with a study of the surface treatment impact on the chemical composition of the surface itself was carried out by means of a Hitachi TM3000 scanning electron microscope (SEM, Hitachi, Tokyo, Japan) equipped with a National Instruments energy dispersive spectroscopy (EDX) microprobe (National Instruments Italy, Assago, Italy). Images with roughly one million pixels were taken, without the use of flash or a filter, each image having a resolution of 1280 × 800 pixels.

Image processing was performed by means of the two-dimensional Fourier descriptors (FD) to decode the image obtained by the SEM [27,28,29]. It was thus possible to study the image as a matrix of gray-scale pixels along three different directions: Horizontal, vertical and diagonal [30,31].

The variables were described with the use of frequencies for categorical variables and the median and range for quantitative variables. To evaluate the relations between the different characteristics of the implant drills a correlation matrix and a repeated measures ANOVA with a Bonferroni post-hoc correction were performed (within/between subjects). All *p* values were two-sided and values <0.05 were considered significant.

The calculations were performed by using MATLAB^®^ Ver. R2015b software (1 Apple Hill Drive Natick, MA 01760-2098, USA). Statistical analysis was performed using the open source software Jamovi (Version 0.9 [Computer Software] (Freely retrieved from https://www.jamovi.org) [32]. Vickers microhardness was measured using a CV 2000 microhardness tester (Bowers Group Testing Istruments, Camberley, UK) with an indentation force of 500 g [33].

## 3. Results

### 3.1. Chemical Composition

The implant drill composition was that typical of martensitic stainless steel (MSS). The chemical composition of each drill is reported in Table 1 below.

The surgical drills contained 13%–17% of chromium (Cr); molybdenum (Mo), silicon (Si) and manganese (Mn) were present as minor ligands. The MSS of all the surgical drills examined were subjected to previous heat treatment, which consists in heating followed by quenching [34]. The steel is heated to a temperature at which the alloy is converted exclusively into austenite. Quenching transforms the austenite into martensite due to a diffusion less transformation. Further, quenching is often performed in order to give the component the desired hardness [34]. Usually, at the end of quenching some residual austenite could be present in the microstructure. The presence of residual austenite causes softening, which makes it highly undesirable in making surgical drills. 

In some surgical drills examined martensite carbide grains were reported. The presence of fine dispersed carbides in a martensitic matrix increases the hardness and wear resistance of the component [34]. To control the formation of carbides Mo is usually added.

#### 3.1.1. Microstructural Conformation

**Figure 1:**
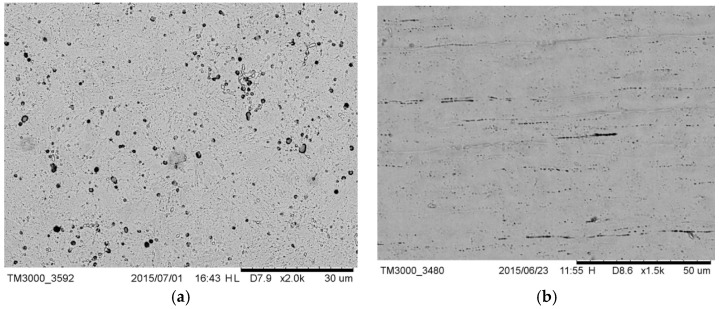
(**a**) Microstructure of drill C containing sulfide precipitates; (**b**) external surface of drill C; milling lines were visible.

The microstructure of drill C (Figure 1a) was lamellar martensite containing some black precipitates identified as manganese sulfide added to increase the machinability of the alloy. The external surface of the drill (Figure 1b) showed the typical horizontal milling lines, as a result of the machining treatment [35]. 

**Figure 2:**
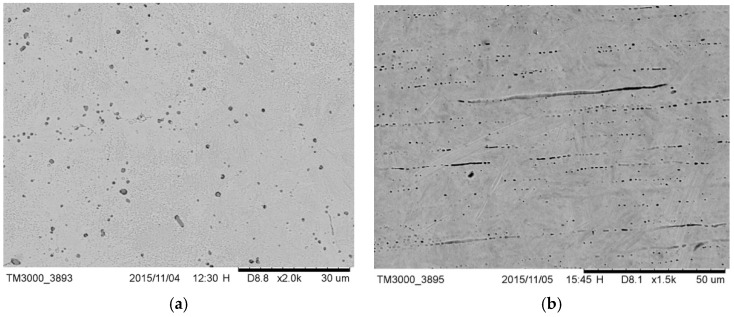
(**a**) Microstructure of drill E where sulfide precipitates were visible; (**b**) external surface of drill E; milling lines were visible.

The microstructure of drill E (Figure 2a) was lamellar martensite containing some black precipitates identified as manganese sulfide added to increase the machinability of the alloy. The external surface of the drill (Figure 2b) also showed horizontal milling lines as a result of the machining treatment. 

**Figure 3:**
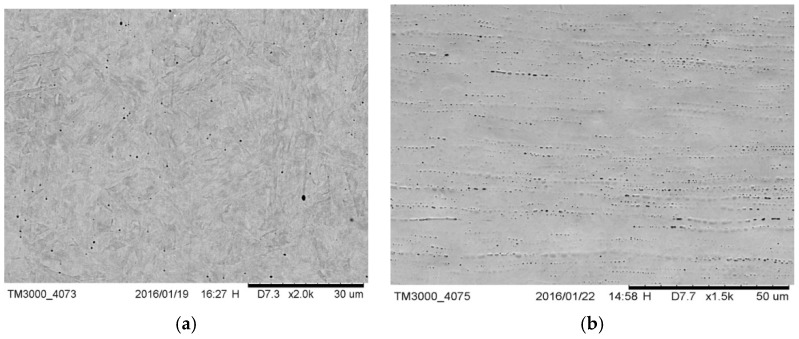
(**a**) Microstructure of drill B; there were no precipitates and (**b**) external surface of drill B; milling lines were visible.

Drill B had a lamellar martensitic structure substantially free from precipitates (Figure 3a). The external ground surface (Figure 3b) was quite similar to that of drills C and E; milling lines were visible. 

**Figure 4:**
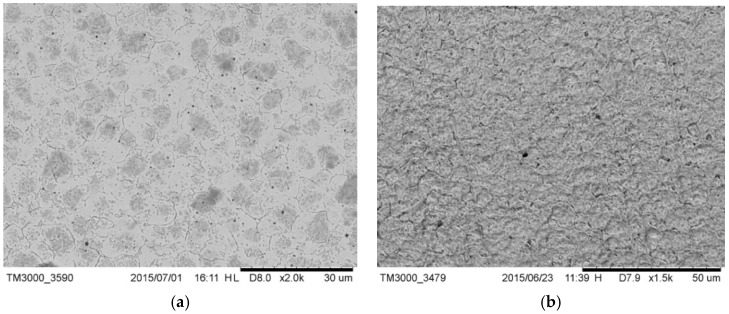
(**a**) Microstructure of drill A; fine dispersed carbides were visible; (**b**) external surface of drill A; the absence of mailing line was evident; it showed prominent irregular ridges.

The microstructure of drill A (Figure 4a) was annealed to the martensite matrix with dispersed fine carbides such as M_23_C inside. The absolute absence of any milling line and of any other evident technological signature of the same kind on the external surface (Figure 4b) leads to the conclusion that the bone drill was manufactured by plastic deformation. The comparison of reported images with those available in literature suggested it [36]. The presence of small, uniformly distributed carbides suggests that annealing was performed to soften the alloy prior to obtaining the final microstructure by means of hot forming. This manufacturing process assured the desired value of hardness. 

**Figure 5:**
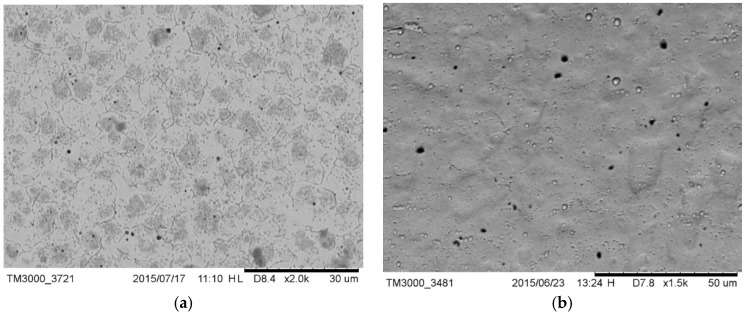
(**a**) Microstructure of drill D; fine dispersed carbides were visible; (**b**) external surface of drill D; some residual porosity was visible.

The microstructure of drill D, illustrated in Figure 5a, also had an annealed martensitic matrix with dispersed carbide particles (i.e., M_23_C). Precipitates were evident even on the external surface characterized by some residual porosity without milling lines (Figure 5b), suggesting that it was manufactured, as the A bone drill, by heat treatment after forming operations.

**Figure 6:**
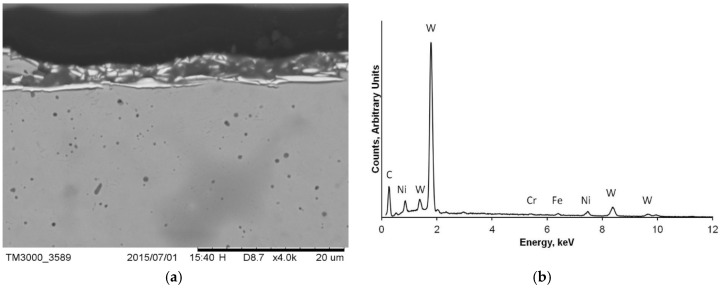
(**a**) Drill D section, which evidenced the tungsten carbide coating; (**b**) energy dispersive spectroscopy (EDX) spectra of the coating of drill D; the presence of W and Ni was predominant.

The cross section of drill D (Figure 6a) showed the presence of a thin coating. The EDX spectrum of the external surface of the dental implant drill, reported in Figure 6b, showed the predominant presence of W and Ni, which indicated the coating was made of hard tungsten carbide.

In none of the implant drills analyzed was the presence of retained austenite observed, which indicates a high quality of the alloys employed and of the heat treatment performed to obtain the desired qualities.

The differences in the microstructure of the drills reflect the difference in the industrial procedures for manufacturing the drills. Drills B, C and E were characterized by the presence of lamellar martensite, which indicates that the final shape was obtained by machining. In the case of C and E, sulfur was added to the alloy to increase machinability. In drill B, the final shape was obtained by choosing a lower value of hardness. Drills A and D were characterized by the presence of annealed martensite, which indicates that the final profile was obtained by means of hot working. 

Table 2 reports the measured hardness for each of the implant bone drills examined. The reported values are typical of martensitic stainless steel (MSS) [33].

#### 3.1.2. Microstructure Obtained by Fourier Descriptors (FD) 

**Figure 7:**
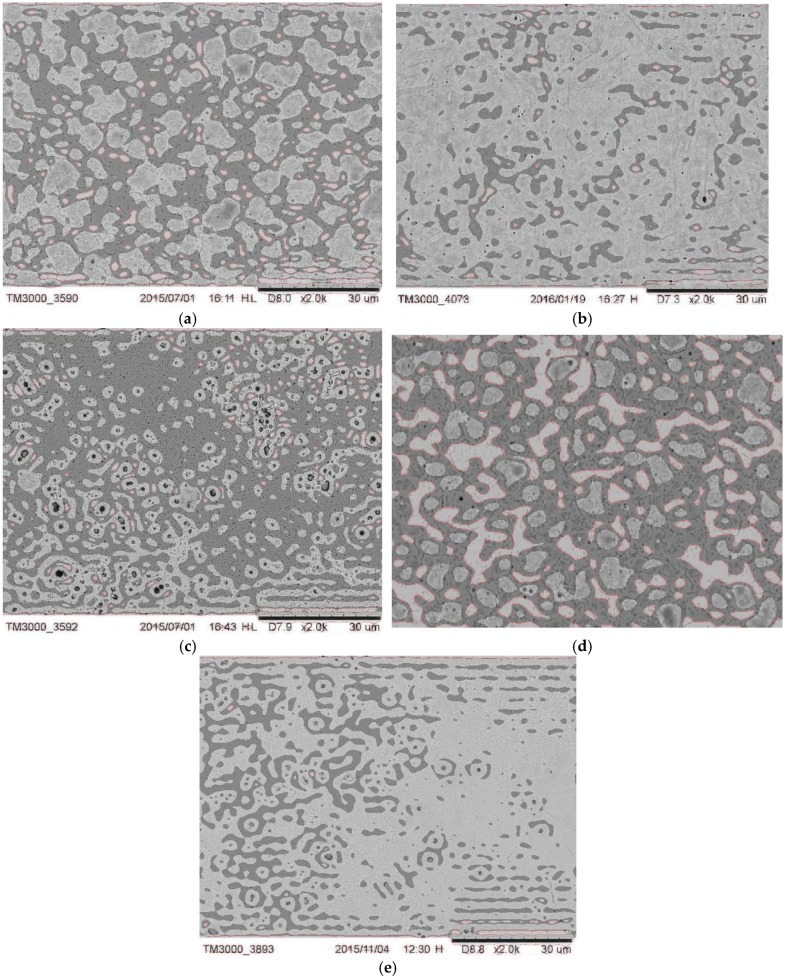
(**a**) Two-dimensional processing image of the A drill microstructure; (**b**) two-dimensional processing image of the B drill microstructure; (**c**) two-dimensional processing image of the C drill microstructure; (**d**) two-dimensional processing image of the D drill microstructure and (**e**) two-dimensional processing image of the E drill microstructure.

FD allowed quantitative measurements to be made on the microstructure image in order to obtain a description and interpret its content [27,28,29]. From image processing of the surgical drill microstructures (Figure 7) some information, such as the contrast, energy, homogeneity and correlation, was obtained [30,31]. Table 3 reports the descriptives for the implant drill characteristics analyzed.

**Figure 8:**
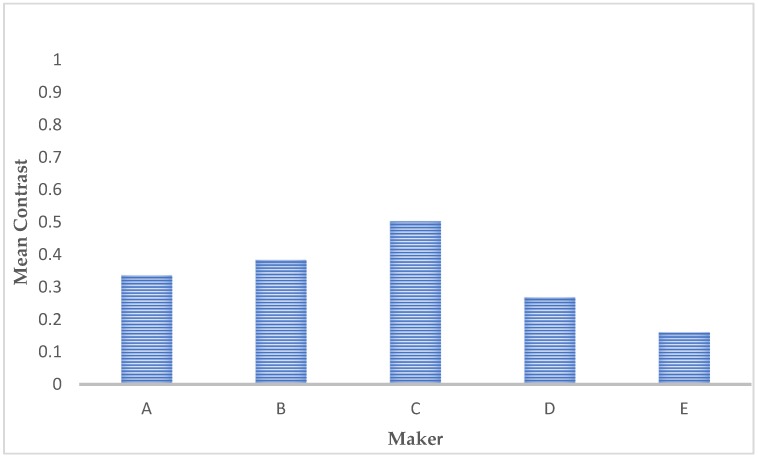
Comparison of the contrast values presented by the different microstructure images.

The average contrast values of the different two-dimensional processing images of microstructures are reported in Figure 8: A low value of contrast means the image has almost constant gray levels, and the relative microstructure can be defined as quite homogeneous. Conversely, a high value means that appreciable metallurgical features, such as precipitate grain boundaries or different phases, characterize the observed microstructure.

**Figure 9:**
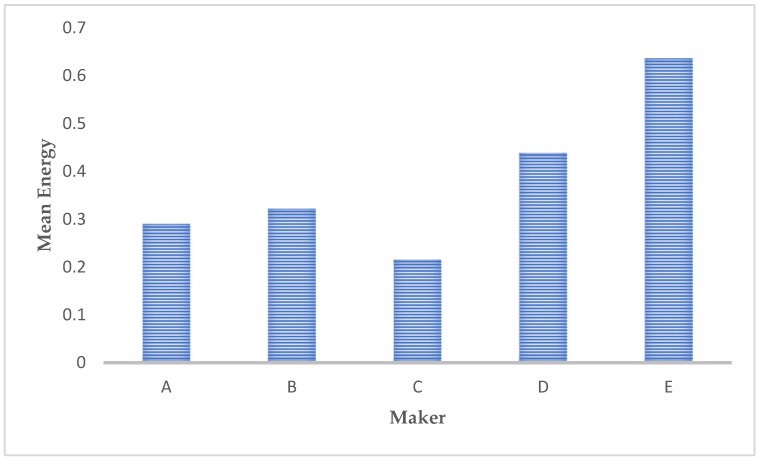
Comparison of the energy values presented by the different microstructure images.

The average energy values of the different microstructure images are reported in Figure 9: Higher values correspond to homogeneous microstructures; this means that the differences in gray values are almost zero in most areas. Low values are found when differences are evenly distributed spatially.

Microstructures with the highest energy values are those with the highest degree of homogeneity; while those with lowest energy values are the microstructures with the most appreciable presence of the above-mentioned metallurgical features.

**Figure 10:**
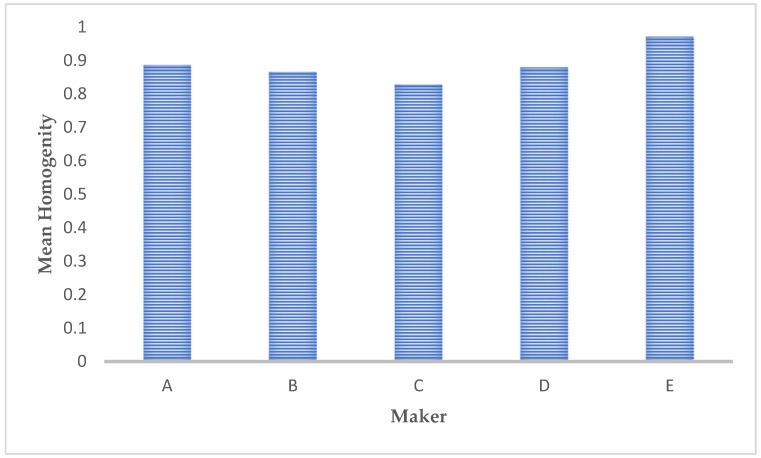
Comparison of the homogeneity values presented by the different microstructure images.

Homogeneity (Figure 10) measures the level of uniformity or roughness (fineness/coarseness) of the observed microstructures, analyzing the repetitive spatial frequencies and, as a consequence, the dimensions of the different structures. Texture characterized by fine structures, shows high values of spatial frequencies; vice versa texture showing coarser structures presents low spatial frequencies. It can be easily appreciated that the greater the energy, the greater the homogeneity.

**Figure 11:**
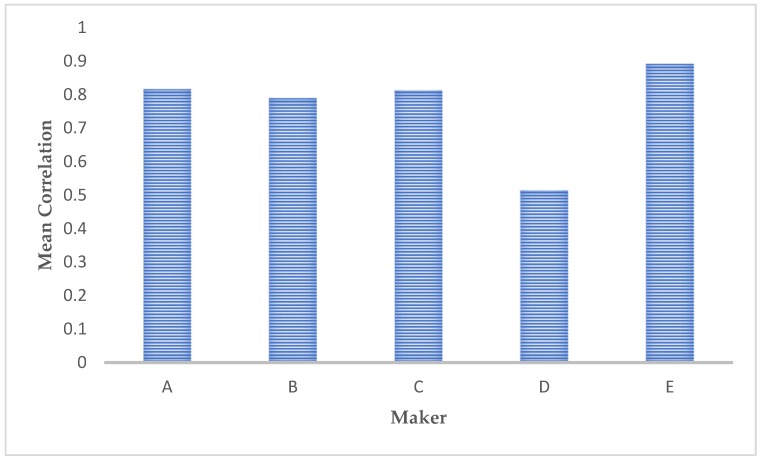
Comparison of the correlation values presented by the different microstructure images.

The spatial relationship between the different structures is described by the correlation coefficient (Figure 11). A low value of correlation means no great distances between the different structures are reported; the latter condition characterizes microstructures with coarser structures. Conversely, a high value means that greater spatial distances between the different structures are evidenced. 

**Figure 12:**
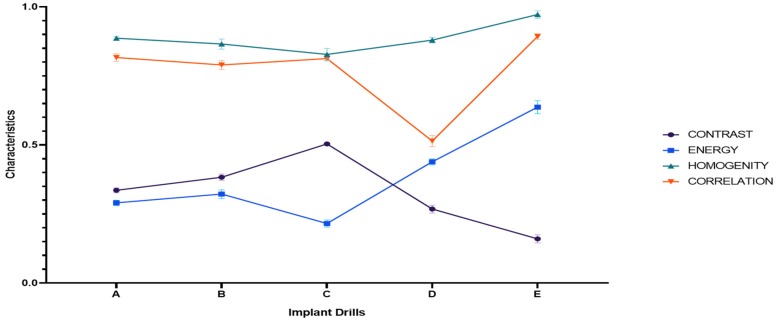
Graphic representation of the mean values of the different characteristics in the implant drills analyzed.

A graphic representation of the measurements for the different characteristics and implant drills is reported in Figure 12.

**Figure 13:**
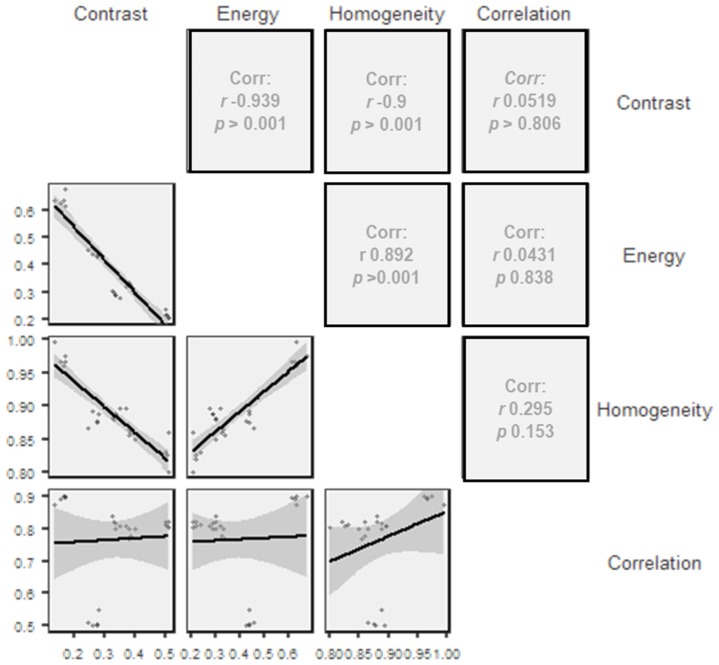
Correlation matrix (Pearson) for the different characteristics analyzed.

As represented in Figure 13, there is an indirect correlation between contrast/energy and contrast/homogeneity, while between homogeneity/energy and homogeneity/correlation there is a direct correlation in the measurements of the different implant drills. For the other pairs, the correlation matrix shows an almost linear distribution of the observations.

To attain a better understanding of the characteristics of the implant drills and their relatedness, a repeated measure ANOVA (RMA), with a post-hoc Bonferroni correction was performed. Table 4 reports the results of the RMA for within (WSE) and between (BSE) analyses. Both the WSE and the BSE report significant differences in the overall measurements for the different characteristics and implant drills analyzed.

## 4. Discussion 

The chemical composition and surface conformation of five different implant bone drills were investigated. All the implant drills examined were made of martensitic stainless steel (MSS); only one was also coated by a thin layer of tungsten carbide (Implant drill D). The MSS shows high hardness leading to high wear resistance and, as a consequence, is generally suitable for producing dental implant drills [32]. Retained austenite, which is the major defect of MSS, was not detected in all the examined dental implant bone drills. Drills B, C and E were characterized by the presence of lamellar martensite. In the case of C and E, sulfur was added to the alloy to increase machinability. In drill B, a lower value of hardness ensured the desired profile. The microstructure of drills A and D was characterized by the presence in plate martensite of dispersed carbides, which increases their hardness.

Hardness, toughness and wear are considered important parameters for evaluating the clinical performance of surgical bone drills [17,25,37]. Hardness is directly linked to wear resistance: The greater the hardness, the higher the wear resistance [15]. It is worth noting that the selected manufacturers have adopted different technical approaches to increase bone drill hardness and cutting power. Implant drills A and D had a higher chromium content, which ensures greater hardness (Table 2) [38]. In implant drill D a thin coating made of tungsten carbide increases the hardness of the external surface [21]. These implant drills also show the potential highest tissue removal rate due not only to their hardness, but also to their surface conformation: Both have a rough external surface that could contribute to bone removal through an abrasive mechanism. This condition could lead to more heat generation during bone drilling [39]. Indeed, Oh et al. showed to what extent the size of the contact area between the drill and the bone affects frictional heat: Increased surface area in contact with the bone results in increased frictional heat [39,40]. Using implant drills B, C and E, formed probably by machining, which allows an accurate shape to be obtained, bone removal should be obtained only by the cutting action performed by the edge on the bone, predisposing to less heat generation. However, because cutting efficiency and, as a consequence, the heat generation were not evaluated, it is difficult to draw any conclusions; it only may be suggested that using implant drills, produced by the machining treatment, with an accurate cutting part, a lower increase in bone temperature should have been the expected outcome respect the implant drills produced by heat treatment. More studies directly correlating the drill superficial conformation with cutting efficiency are needed to draw a conclusion.

Wang et al. reported that the heat generated by cutting is due to the energy released in response to the very large deformation involved in cutting chips of the bone [41]. 

The ability of the bone drill to adsorb energy and plastically deform without fracturing (toughness) is another important feature [42,43]. It increases as hardness and wear resistance decrease [24,25]. The hardness values of implant drills B and C evidence their greater predisposition to plastic deformation during bone cutting that could result in decreased frictional heat. Eriksson and Albrektsson found that temperatures of 47 °C generated for one minute at the bone interface will induce osteonecrosis locally, which can compromise implant osseointegration [44]. Bone necrosis occurs as a result of many intracellular changes: Protein denaturation, inactivation of enzymes for cell metabolism, alteration in protoplasmic lipids, cell dehydration, membrane rupture and finally carbonization. Generated heat also causes dislocation in the hydroxyapatite mineral lattice structure and microscopic creeps of the compact bone [45]. Since implant success requires remodeling of the bone around the surgical site without interposition of the connective tissue, heat generation during the preparation of the recipient site is cited as a major factor influencing implant failure [25,39,46]. 

Drill wear is another important factor in heat generation during bone drilling [11,22,25]. It is an unavoidable and irreversible process, which increases friction in the cutting zone [47]. Besides a negative thermal impact, wear causes higher cutting forces and a drill vibration, which can result in cutting edge breakage, or complete drill breakage in the flute or shank zone [48,49]. Implant drills A and D showing a high hardness could also ensure a high wear resistance. By contrast, drills B, C and E have lower wear resistance: Their repeated use could decrease their cutting efficiency and increase frictional heat generation.

The Fourier analysis technique is widely used in image processing to describe and classify shapes. In our case, it was also useful to quantify the differences between the examined microstructures [27,28,29,30,31]. The reported contrast and energy values showed that implant drills A, B and C are characterized by more appreciable metallurgical features than bone drills E and F. Homogeneity evaluation evidenced that bone drills A and E had finer structures than B, C and D. A homogeneous microstructure made of fine grains is correlated to greater hardness and wear resistance [50]. These considerations were in accordance with the correlation evaluation, which showed that bone drills A and E presented more spatial distance between the different structures of texture with respect to the other implant bone drills examined.

Repeated measures ANOVA analysis reported a significant difference both within the overall implant drill observations and in comparing the characteristics observed for each implant drill, meaning that the observations recorded for each implant drill are distinguished from each other and dependent on the implant drill analyzed. Similarly, in the between subject effects the analysis showed a significant overall difference between implant drills in the observations recorded for the characteristics analyzed. The construction of the correlation matrix highlighted the linear correlation between the different characteristics of the implant drills. Especially for the pairs energy/contrast, homogeneity/contrast and energy/homogeneity there is a strong correlation (>0.7) while for the other pairs a weak correlation was reported (<0.3). Furthermore, for the homogeneity measurements there was an almost linear distribution between implant drills A, B, C and D. Implant drill D is the one that differs most in the values for the correlation parameter, while energy and contrast differ the most between the drills.

The composition of the implant drills examined was typical of martensitic stainless steel (MSS). Different treatments had been used to improve the hardness and wear resistance of the external layer of the dental implant drills. The Straumann and Global D implant drills, produced by hot forming, showed higher values of hardness, but their surface microstructure could lead to a greater amount of heat being generated during bone drilling. By contrast, NobelBiocare, Friadent and Sweden & Martina, produced by machining, showed lower values of hardness; their more accurate shapes predispose them to less heat generation during implant site preparation. 

## Figures and Tables

**Table 1:** Chemical composition of the different dental implant drills examined.

Implant Drill	Fe (wt %)	Cr (wt %)	Mn (wt %)	Si (wt %)	Mo (wt %)
A	Balance	17.0	0.6	0.6	0.7
B	Balance	13.4	1.4	0.4	0.4
C	Balance	13.7	1.2	0.6	0.4
D	Balance	17.1	0.8	0.5	0.6
E	Balance	13.1	1.2	0.5	0.5

**Table 2:** Vickers micro-hardness of the dental implant drills examined. The values are those typical of steel employed to make surgical drills [32].

Implant Drill	Hardness
A	600.3
B	466.6
C	530.2
D	674.8
E	589.1

**Table 3:** Descriptives for the contrast, energy, homogeneity and correlation values of the implant drill microstructure images examined.

Group Descriptives					
	Implant Drills	N	Mean	SD	SE
Contrast	A	5	0.336	0.00890	0.00398
	B	5	0.383	0.01123	0.00502
	C	5	0.504	0.00509	0.00227
	D	5	0.268	0.01411	0.00631
	E	5	0.160	0.01456	0.00651
Energy	A	5	0.290	0.00990	0.00443
	B	5	0.322	0.01545	0.00691
	C	5	0.216	0.01303	0.00583
	D	5	0.439	0.01106	0.00495
	E	5	0.637	0.02369	0.01059
Homogeneity	A	5	0.886	0.00677	0.00303
	B	5	0.865	0.01826	0.00817
	C	5	0.828	0.02141	0.00957
	D	5	0.880	0.01040	0.00465
	E	5	0.972	0.01360	0.00608
Correlation	A	5	0.817	0.01390	0.00622
	B	5	0.790	0.01615	0.00722
	C	5	0.813	0.00837	0.00374
	D	5	0.514	0.02034	0.00910
	E	5	0.893	0.01031	0.00461

**Table 4:** Repeated measures ANOVA (RMA) within/between subjects effects.

**Within Subjects Effects (WSE)**						
	**Sum of Squares**	***N***	**df**	**Mean Square**	**F**	***p***
Implant Drills characteristics	57.446	4	3	19.149	9311	<0.001
Implant Drills characteristics * Implant Drills	11.499	25	12	0.0958	466	<0.001
Residual	0.0123	100	60	2.06 × 10^−4^		
**Between Subjects Effects (BSE)**						
Implant Drills	0.19926	5	4	0.0498	270	<0.001
Residual	0.00369	25	20	1.84 × 10^−4^		

Note. Type 3 Sums of Squares. * Dependence of Implant Drills Characteristics on Implant Drills types. Specifies the correlation between the observed characteristics and the type of implant drill.
